# Behavioral Profiles of Exam Stress: Mapping Student Diet, Sleep, and Substance Use

**DOI:** 10.3390/nu18172847

**Published:** 2026-08-31

**Authors:** Andreea-Sabina Butucaru, Iustina-Gabriela Mihăianu, Magdalena Iorga, Lucian Roman Șipoș, Dan Alexandru Slăvescu, Raluca Ortensia Cristina Iurcov

**Affiliations:** 1 Faculty of Medicine, “Grigore T. Popa” University of Medicine and Pharmacy, 700115 Iasi, Romania; nutr-rom-487@students.umfiasi.ro (A.-S.B.); nutr-rom-514@students.umfiasi.ro (I.-G.M.); 2 Faculty of Psychology and Educational Sciences, “Alexandru Ioan Cuza” University, 700554 Iasi, Romania; 3 Faculty of Medicine and Pharmacy, University of Oradea, 410087 Oradea, Romaniaslavescudan@uoradea.ro (D.A.S.); riurcov@uoradea.ro (R.O.C.I.)

**Keywords:** emotional eating, cognitive restraint, uncontrolled eating, sleep quality, university students, lifestyle, smart drugs, caffeine, smoking behavior, physical activity

## Abstract

**Background**: Academic pressure during examination periods is frequently reported alongside irregular lifestyle behaviors. The study aimed to explore the interplay between habitual eating behaviors, sleep quality, and self-reported stimulant use and coping adaptations specifically during examination periods among healthcare university students. **Methods**: This cross-sectional study included 265 university students (Mage = 22.00, SD = 4.46). Dietary behaviors and sleep quality were assessed using the Three-Factor Eating Questionnaire (TFEQ-R21) and the Pittsburgh Sleep Quality Index (PSQI), respectively. Behavioral profiles, substance-use patterns, and attitudes toward academic pressure were examined using bivariate analyses, multivariate logistic regression, and TwoStep cluster analysis. Statistical analyses were performed using IBM SPSS Statistics version 26.0. **Results**: A high prevalence of poor sleep quality was observed, with 72.8% (*n* = 193) of participants meeting the clinical criterion for poor sleep (PSQI > 5.0; M = 7.79 ± 3.53), and high sleep disturbances were significantly associated with smoking, energy drink use, coffee escalation, general dietary supplement utilization, and smart-drug use. Multivariate analysis revealed that poor sleep quality (OR = 1.118, 95% CI [1.022, 1.222]) and younger age (OR = 0.843, 95% CI [0.735, 0.967]) were independently associated with higher odds of energy drink use. A distinct cognitive disconnect was observed in tobacco usage: conventional smoking was negatively associated with self-perceived health (OR = 0.401, 95% CI [0.182, 0.884]), whereas electronic cigarette use showed no significant association. TwoStep cluster analysis identified three distinct student typologies during exams: non-users (32.6%), poly-substance users (34.5%), and OTC supplement/caffeine users (33.0%), with active substance-utilizing profiles displaying significantly worse sleep quality and higher emotional eating. **Conclusions**: These findings highlight distinct cross-sectional lifestyle and coping patterns among healthcare students, emphasizing the need for targeted institutional interventions that support healthy behavioral adaptations and stress management during academic challenges.

## 1. Introduction

The intersection of psychological distress, sleep disturbances and dietary habits among university students remains a critical focus of public health research. The transition from secondary education to university represents a major developmental shift, exposing students to new academic, social, and personal challenges. These difficulties may be particularly pronounced among students who move away from their families and must adapt to greater independence. Within healthcare disciplines, such challenges are further intensified by the demanding nature of the curriculum. Medical and healthcare students are required to cope with long degree programs, heavy daily schedules, and the simultaneous integration of theoretical coursework with clinical training. In addition, their academic calendars are often extended, with longer semesters than those typically found in other university programs.

In addition to these structural demands, healthcare students face specific psychological stressors, including the emotional burden associated with interactions in clinical and patient-care contexts. Continuous exposure to academic pressure, clinical responsibilities, and emotionally demanding situations may increase their vulnerability to high levels of stress, anxiety, and burnout [1,2].

These risks tend to become especially pronounced during examination periods, which represent particularly challenging moments for students’ overall well-being.

Dietary habits and sleep patterns are among the dimensions most strongly affected during examination periods. Under exam-related pressure, medical students frequently report poorer sleep quality and unfavorable changes in eating behavior. A large amount of research has highlighted an increase in maladaptive behaviors during exam sessions, including irregular eating patterns, night eating, and greater reliance on coffee or energy drinks to maintain academic performance [3,4,5,6]. Previous studies showed that healthcare students also exhibited a high rate of smart-drug consumption to improve mental performance, focus, memory, motivation, and wakefulness and included both prescription drugs, off-label or natural and over-the-counter substances (such as herbal extracts, neuroprotective agents, vitamins and essential minerals, amino acids and metabolic precursors, etc.) [7,8].

Understanding these interconnected vulnerabilities is essential for designing targeted institutional interventions aimed at protecting the physical and mental health of future healthcare professionals.

The scientific literature offers diverse, interesting, and sometimes contradictory results regarding health-related behaviors among healthcare students going through academic assessment periods [9,10]. While the existing literature has extensively documented the individual impacts of academic stress on students’ sleep, dietary habits and stimulant consumption, previous research within medical and healthcare education has predominantly relied on variable-centered approaches that evaluate these parameters in isolation. Michels et al. [11] conducted a study among Belgian students and found that overall diet quality declined during the examination period. This decline was reflected in a lower general diet quality index, reduced fruit and vegetable intake, increased fast-food consumption, and greater difficulty maintaining a healthy diet. The study further showed that certain groups were more vulnerable to exam-related dietary deterioration, including emotional eaters, external eaters, individuals with a preference for sweet and fatty foods, those who considered health an important food-choice motive, participants sensitive to reward or punishment, highly sedentary individuals, non-first-year students, and those reporting high levels of stress.

In a study conducted among Finnish university students, El Ansari et al. [12] found that stress was associated with a lower perceived importance of healthy eating, regardless of gender and BMI. However, stress was not significantly related to the consumption of sweets, cookies, or snacks. These findings suggested that although stressed students may place less importance on nutrition, stress does not necessarily lead to an increased preference for fast food or snack foods. Nevertheless, the scientific literature indicates that the relationship between nutrition and stress can be explained through physiological mechanisms, as stress may directly influence the motivation to eat.

Saddique et al. [13] found strong links between high stress and a Western snack-heavy profile. Also, poor dietary habits were observed among Saudi Arabian medical students, with approximately 66% of respondents reporting skipping breakfast, while 69% indicated frequent consumption of unhealthy snacks. In addition, 61.5% reported eating fruits fewer than three times per week and 82.3% consumed less than 2 liters of drinking water per day [14].

Jamil et al. [15] identified that Turkish medical students often skipped breakfast, with 40.0% of participants reporting that lack of appetite or limited opportunities to eat are the main reasons. Most respondents (95.2%) did not follow a specific dietary pattern. Protein- and carbohydrate-rich foods were commonly preferred, particularly among males (96.2%) and females (75.3%), while females reported a higher preference for fruits and vegetables.

Bitar et al. [16] found that Syrian senior medical university students consumed lower amounts of tea, instant coffee and fast food than freshman students. The researchers also identified that the number of smokers was found to be lower during the exam period than outside the exam period for both cigarettes and e-cigarettes.

In Spain, Mudarra-García et al. [17] suggested that dietary deterioration and reduced physical activity affect healthcare students’ body composition during the examination periods.

In Nepal, Paudel et al. [18] showed that senior medical students exhibited poor sleep quality during exam season, especially those who consumed alcohol and those with low academic grades.

In Poland, Kosendiak et al. [19] showed that the use of sleep medications was highest among medical students, and consumption increased for all students, except those with the highest nutritional knowledge, who exhibited the best sleep.

The scientific literature provides results on the eating and sleeping patterns of medical science students (as opposed to those of students from other faculties), but few studies mention these temporary changes during exam periods. Although several studies have examined eating habits and sleep patterns among medical students in Romania, no research has specifically explored students’ self-reported coping behaviors and perceived consumption patterns during examination periods [20,21,22,23].

The research aimed to bridge the gap between environmental stressors and behavioral patterns. The principal goal of the present research is to simultaneously assess diet, sleep and stimulant use under academic stress rather than by relying on isolated variables. By applying data-driven clustering, the study identifies distinct, multi-dimensional behavioral profiles that traditional single-variable analysis overlook. This study does not focus on individual metrics, but on the synergic integration of this specific setting, simultaneous multi-behavioral tracking and quantitative profile clustering, offering a complete framework for understanding students’ lifestyle choices during critical evaluation periods. The study also considers the ethical dimensions of smart-drug consumption during examination sessions through self-disclosure items.

Accordingly, the hypotheses of the present study, which form part of a broader research project, are as follows:

**H1.**  *The use of exogenous stimulants and performance enhancers as coping strategies during periods of high academic demand is associated with increased global sleep disturbances.*

**H2.**  *Positive health behaviors, such as daily fruit consumption and regular physical activity, are positively associated with cognitive dietary restraint and lower sleep disturbances.*

**H3.**  *Maladaptive eating schedules, such as skipping main meals or not maintaining a regular three-meal routine, are associated with higher levels of emotional and uncontrolled eating, whereas intentional meal omission is related to greater dietary self-monitoring.*

**H4.**  *Poor sleep quality and younger age are significant independent factors associated with higher odds of energy drink consumption during examination periods, whereas psychometric eating behaviors do not appear to exhibit independent associations with this reliance.*

**H5.**  *Students’ self-assessment of maintaining a healthy lifestyle during periods of high academic demand is positively associated with nutritious dietary habits (daily fruit intake, regular meals) and negatively associated with tobacco use, fast-food consumption, and physical inactivity.*

**H6.**  *The likelihood of using smart drugs during examination periods is associated with a combination of students’ academic pressure-related justifications, ethical perceptions, and perceived professional responsibilities.*

## 2. Materials and Methods

### 2.1. Participants and Study Design

An observational, cross-sectional study was conducted between November 2025 and May 2026 among undergraduate health sciences students from two Romanian academic centers: “Grigore T. Popa” University of Medicine and Pharmacy (Iași) and the Faculty of Medicine, University of Oradea. A non-probability snowball sampling strategy was employed via digital channels (institutional student groups, WhatsApp, and academic social networks) using student representatives as primary seeds.

Participation was voluntary, anonymous, and unpaid. Because the recruitment invitation was shared openly across digital platforms, the total number of exposed individuals could not be objectively recorded; therefore, a formal response rate cannot be determined. Duplicate and non-consenting submissions were removed, yielding a final cohort of *n* = 265 valid cases.

Of the participants, a total of 163 students (61.5%) were enrolled at the “Grigore T. Popa” University of Medicine and Pharmacy Iași and 38.5% at the University of Oradea (*n* = 102). In terms of academic progression, the majority were in their preclinical training years (80.4%, *n* = 213; Year 1: 40.4%, Year 2: 6.8%, Year 3: 33.2%), while 18.1% were in their clinical training years (*n* = 48; Year 4: 5.7%, Year 5: 8.3%, Year 6: 4.2%), and 1.5% (*n* = 4) were enrolled in master/allied health tracks. Female students represented 74.3% of the sample (*n* = 197), reflecting the known demographic feminization in Romanian medical higher education, although voluntary participation bias cannot be excluded [20,21,22,23].

### 2.2. Research Tool

Data collection was executed using a self-administered online questionnaire hosted on the Google Forms platform (Alphabet, Mountain View, CA, USA). The measurement instrument was structured into four main functional sections:

- Socio-demographic profile and lifestyle characteristics: This section recorded baseline parameters including age, sex, residential background (urban vs. rural), accommodation status during university studies (rented apartment, university dormitory, or living with parents), academic discipline, and current study year. Dietary patterns were evaluated by recording adherence to a regular three-meal daily schedule, specific meal-skipping habits (omission of breakfast, lunch, or dinner), inter-meal snacking, late-night eating (after 21:00), daily fruit consumption (≥1 serving/day), and weekly intake frequency of fast food and carbonated soft drinks (assessed via 4-point ordinal frequency scales). Lifestyle behaviors were captured by assessing smoking status (distinguishing non-smokers, former smokers, and active users of conventional cigarettes vs. electronic vaping devices), alcohol consumption frequency, daily physical activity (≥30 min/day), and predominant exercise intensity (light, moderate, or intense). Self-perceived healthy-lifestyle status, prior history of weight-loss diets, and interest in campus nutritional education programs were recorded using binary items.
- Psychological eating behaviors (TFEQ-R21): This section q eating behavior dimensions using Three-Factor Eating Questionnaire (TFEQ-R21) developed by Karlsson et al. [24]. The tool has 21 items and measures three independent constructs: cognitive restraint (6 items assessing intentional food restriction), uncontrolled eating (9 items measuring loss of satiety control), and emotional eating (6 items assessing affect-triggered food intake). Items were rated on a uniform 5-point Likert response scale ranging from 1 (“Never/Definitely False”) to 5 (“Always/Definitely True”). Domain scores were computed as continuous arithmetic means of the respective items (ranging from 1.00 to 5.00), with higher scores reflecting greater manifestation of each eating pattern. For descriptive categorization and sample visualization, mean scores were divided into equal-interval tertile brackets: low (1.00–2.33), moderate (2.34–3.66), and high (3.67–5.00), representing sample-specific descriptive divisions rather than clinical diagnostic thresholds.
- Subjective sleep quality (PSQI): Operationalized nocturnal sleep quality and sleep architecture using the standard 19-item Pittsburgh Sleep Quality Index (PSQI) developed by Buysse et al. [25], which has been previously validated and widely utilized in Romanian university cohorts [26,27]. The scale evaluates seven diagnostic components (subjective sleep quality, sleep latency, sleep duration, habitual sleep efficiency, sleep disturbances, use of sleep medication, and daytime dysfunction), each rated from 0 to 3. Component scores were aggregated into a global PSQI score (0–21 points), with values exceeding the established clinical cutoff threshold of 5.0 (>5.0) identifying “poor sleepers”.
- Academic coping mechanisms and cognitive enhancement frameworks: Evaluated retrospective, self-reported dietary adaptations, stimulant intake, and ethical frameworks specifically utilized during high-demand examination periods. Self-developed items evaluated students’ perceived increases in the consumption frequencies of coffee, energy drinks, over-the-counter dietary supplements (vitamins, lecithin, ginkgo biloba, and ginseng), and smart drugs as situational coping strategies, distinguishing these exam-specific adaptations from baseline habitual lifestyle measures. To avoid conceptual overlap, supplement use was differentiated into two distinct variables: (1) general dietary supplements (routine broad nutritional and multivitamin/mineral supplementation); and (2) specific over-the-counter (OTC) memory/cognitive supplements (specifically evaluating targeted non-prescription nootropic and herbal formulations, such as lecithin, ginkgo biloba, ginseng, and neuro-support vitamins utilized to enhance exam performance). Specific items evaluating academic pressure justifications, ethical attitudes, and professional obligations regarding smart-drug use were designed by the authors based on the current literature on academic cognitive enhancement. Items were formulated on a standard 5-point Likert scale (1 = “Strongly Agree” to 5 = “Strongly Disagree”) and reviewed by the multidisciplinary research team for face and content validity prior to administration.

### 2.3. Statistical Analysis

Statistical analyses were performed using IBM SPSS Statistics (version 26.0; IBM Corp., Armonk, NY, USA). Continuous variables were expressed as means (M) ± standard deviations (SD) or medians (Mdn) with interquartile ranges (IQR), while categorical parameters were presented as absolute frequencies and percentages (N, %). Due to optional or non-mandatory survey items, minor data incompleteness occurred across certain continuous and categorical variables. Missing values were addressed using pairwise deletion to maximize data retention and analytical power for each specific test. Consequently, the effective sample size varies slightly across sub-analyses (ranging from *n* = 261 to *n* = 265), and exact valid sample sizes are explicitly reported for each individual statistical model and table. Sample size adequacy was verified using G*Power (v. 3.1.9.7), confirming that *n* = 265 provides adequate statistical power (>0.80) for multivariable logistic regression with up to 5 predictors. Model stability was supported by the Events Per Variable (EPV ≥ 10) rule, with 35 positive events across 3 predictors in the smart-drug model (EPV = 11.67).

Data normality was evaluated using the Kolmogorov–Smirnov test. Because continuous metrics (including age, sleep duration, global PSQI scores, and TFEQ-R21 subscale metrics) departed significantly from a normal distribution (*p* < 0.05), non-parametric inferential methods were applied. Bivariate relationships between continuous or ordinal variables were assessed using Spearman’s rank correlation coefficient (r_s_). Proportional disparities across demographic and lifestyle subgroups were tested using Pearson’s chi-square (chi^2^) tests of independence.

Group comparisons across non-normally distributed continuous outcomes were conducted using the Mann–Whitney U test for two-group analyses (e.g., gender, residential background, active smoking, daily physical activity) and the Kruskal–Wallis H test for comparisons involving three or more independent groups (e.g., physical activity intensity levels, meal-skipping patterns). Significant Kruskal–Wallis analyses were followed by pairwise post hoc comparisons using Mann–Whitney U tests. Effect sizes for Mann–Whitney U tests were calculated using the rank-biserial correlation r = $\frac{\left|z\right|}{\sqrt{N}}$, interpreted according to standard benchmarks: small (0.10 ≤ r < 0.30), medium (0.30 ≤ r < 0.50), and large (r ≥ 0.50). For Kruskal–Wallis H tests, effect sizes were estimated using eta-squared based on the H-statistic (ƞ^2^_H_ = $\frac{H-k+1}{N-k}$), interpreted as small (0.01 ≤ ƞ^2^_H_ < 0.06), moderate (0.06 ≤ ƞ^2^_H_ < 0.14), and large (ƞ^2^_H_ ≥ 0.14).

Internal consistency and psychometric reliability for the PSQI components and TFEQ-R21 subscales were established using Cronbach’s alpha coefficient (α). To identify behavioral typologies regarding substance use during examination periods, a TwoStep cluster analysis was performed on binary indicators (coffee escalation, energy drinks, dietary supplements, OTC vitamins, and smart drugs) using a log-likelihood distance measure and Schwarz’s Bayesian Criterion (BIC). Cluster quality was confirmed via the silhouette measure of cohesion and separation. Cross-tabulations were used to describe cluster characteristics, while independent external criterion validity was established by comparing sleep quality (PSQI) and eating behaviors (TFEQ-R21) across clusters using Kruskal–Wallis H tests.

Multivariate associations were modeled using three binary logistic regression analyses to evaluate factors linked to: (1) energy drink reliance during exam periods; (2) pharmacological cognitive-enhancer (“smart-drug”) utilization; and (3) self-perceived healthy-lifestyle status. Candidate predictors were selected based on theoretical relevance and preliminary bivariate significance (*p* < 0.05), with categorical inputs dummy-coded (0 = reference, 1 = presence). Multicollinearity was ruled out prior to model fitting, with Variance Inflation Factor (VIF) values remaining strictly below 2.0. Candidate predictors were selected based on a theoretical framework of academic coping mechanisms. Model fit and performance were evaluated using Omnibus tests, Hosmer–Lemeshow goodness-of-fit tests, and pseudo-R^2^ statistics. To prevent misinterpretation of overall accuracy in unbalanced outcomes, classification sensitivity, specificity, and discrimination via the Area Under the Receiver Operating Characteristic Curve (AUC-ROC with 95% CI) were reported. Results are reported as unstandardized coefficients (Bs), standard errors (SEs), Wald statistics, odds ratios (ORs), and 95% confidence intervals (CIs). Statistical significance was set at a two-tailed *p* < 0.05. To control multiplicity and mitigate the risk of Type I error arising from extensive non-parametric and bivariate and pairwise post hoc testing, the Benjamini–Hochberg False Discovery Rate (FDR) procedure was systematically applied across all 66 bivariate comparisons at a threshold of q = 0.05. Both unadjusted two-tailed *p*-values and FDR-adjusted q-values are reported throughout the results, with q < 0.05 defining statistical significance after multiplicity control. Multivariable binary logistic regression models served as the prespecified primary inferential framework, whereas bivariate tests were treated as secondary and exploratory.

### 2.4. Ethical Approval

The research adhered to the ethical principles set out in the Declaration of Helsinki. The study was approved by the Research Ethics Committee of the University of Oradea, Romania (No. 21129/22 December 2025).

## 3. Results

### 3.1. Socio-Demographic and Lifestyle Data

The study included a sample of *n* = 265 students, with a mean age of M = 22.00 (SD = 4.46 years, ranging from 18 to 47 years), most of them being female (74.3%) and living in urban areas (61.9%). Academically, 61.5% were recruited from Iași (*n* = 163) and 38.5% from Oradea (*n* = 102), with 80.4% (*n* = 213) in preclinical study years and 18.1% (*n* = 48) in clinical study years.

Dietary-habit evaluations revealed that only a small proportion of students regularly consume three meals per day (20.8%), with the vast majority (79.2%) reporting irregular meal patterns. Furthermore, daily fruit consumption was reported by less than half of the sample (48.3%).

Regarding substance use and lifestyle choices, 30.2% of the respondents identified as active smokers—with a preference for electronic cigarettes (22.3%) over conventional ones (18.5%). These categories were not mutually exclusive, as a subset of participants engaged in dual use (concurrent utilization of both conventional and electronic devices).

Although more than half of the respondents (57.7%) perceived their overall lifestyle as unhealthy, 65.3% engaged in at least 30 min of physical activity daily, predominantly of light intensity (56.6%). The health-related behavior and lifestyle characteristics of the sample are systematically presented in Table 1.

The questionnaire collected data on dietary changes during exam periods. The items investigated the consumption of coffee, energy drinks, smart drugs and supplements. Also, a series of items aimed to identify the students’ opinion on the consumption of smart drugs during stressful periods of the academic year. While habitual smart-drug use was reported by 5.3% (*n* = 14) of students, situational use specifically during examination periods was reported by 13.4% (*n* = 35). While the total cohort comprised *n* = 265, a valid analytical sample of *n* = 261 completed this situational module (*n* = 4 cases had missing items and were excluded). Details are presented in Table 2.

### 3.2. Three-Factor Eating Questionnaire (TFEQ-R21)

Eating behavior characteristics were assessed using the three subscales of the TFEQ-R21: cognitive restraint (CR), uncontrolled eating (UE), and emotional eating (EE). All subscales demonstrated good-to-excellent internal consistency, with Cronbach’s alpha coefficients ranging from 0.879 to 0.948. Descriptive metrics and severity classifications for each subscale are summarized in Table 3.

**Figure 1:**
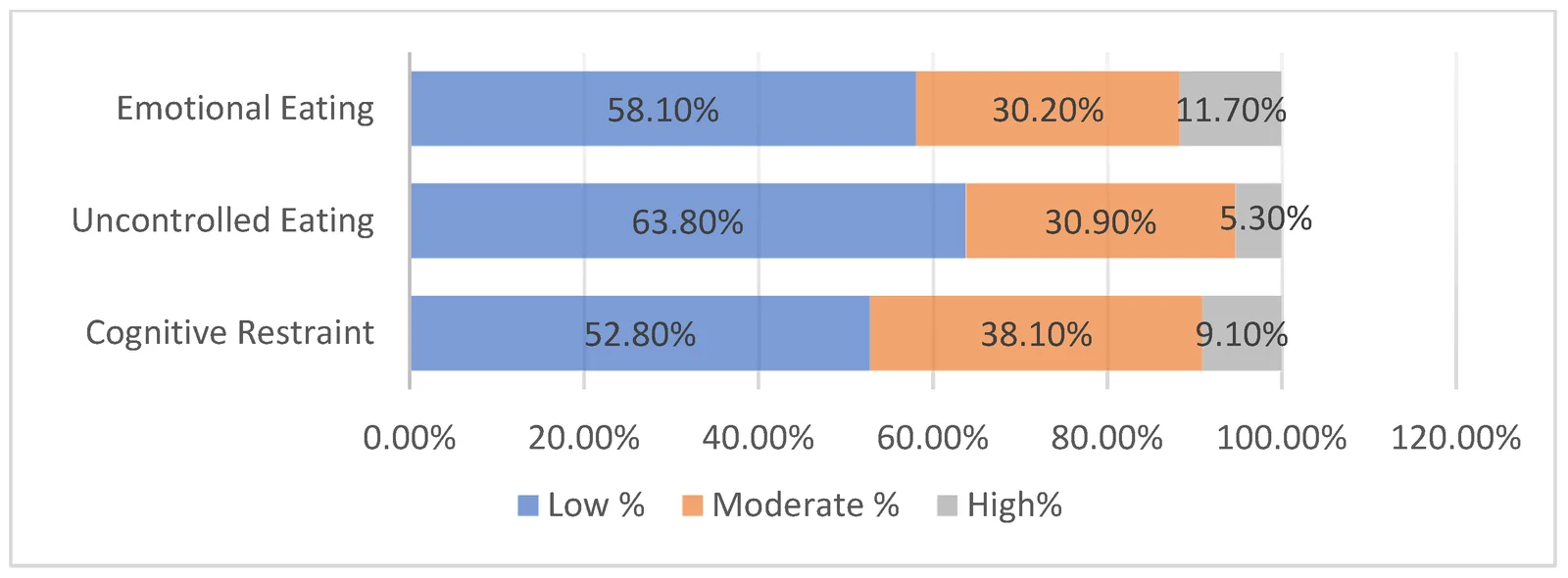
Percentage distribution of participants across severity levels (low, moderate, high) for the TFEQ-R21 eating behavior subscales (*n* = 265).

Across all three eating domains, the majority of participants fell into the low-severity category (>50%). High behavioral manifestation was least prevalent, ranging from 5.3% for uncontrolled eating to 11.7% for emotional eating (Figure 1).

### 3.3. Pittsburgh Sleep Quality Index (PSQI)

To assess the participants’ sleep quality and potential nocturnal disturbances, the standard Pittsburgh Sleep Quality Index (PSQI) was utilized. Psychometric evaluation of the instrument at the sample level (*n* = 265) demonstrated a modest internal consistency (Cronbach’s α = 0.683), which falls slightly below the conventional 0.70 threshold and warrants cautious interpretation.

**Figure 2:**
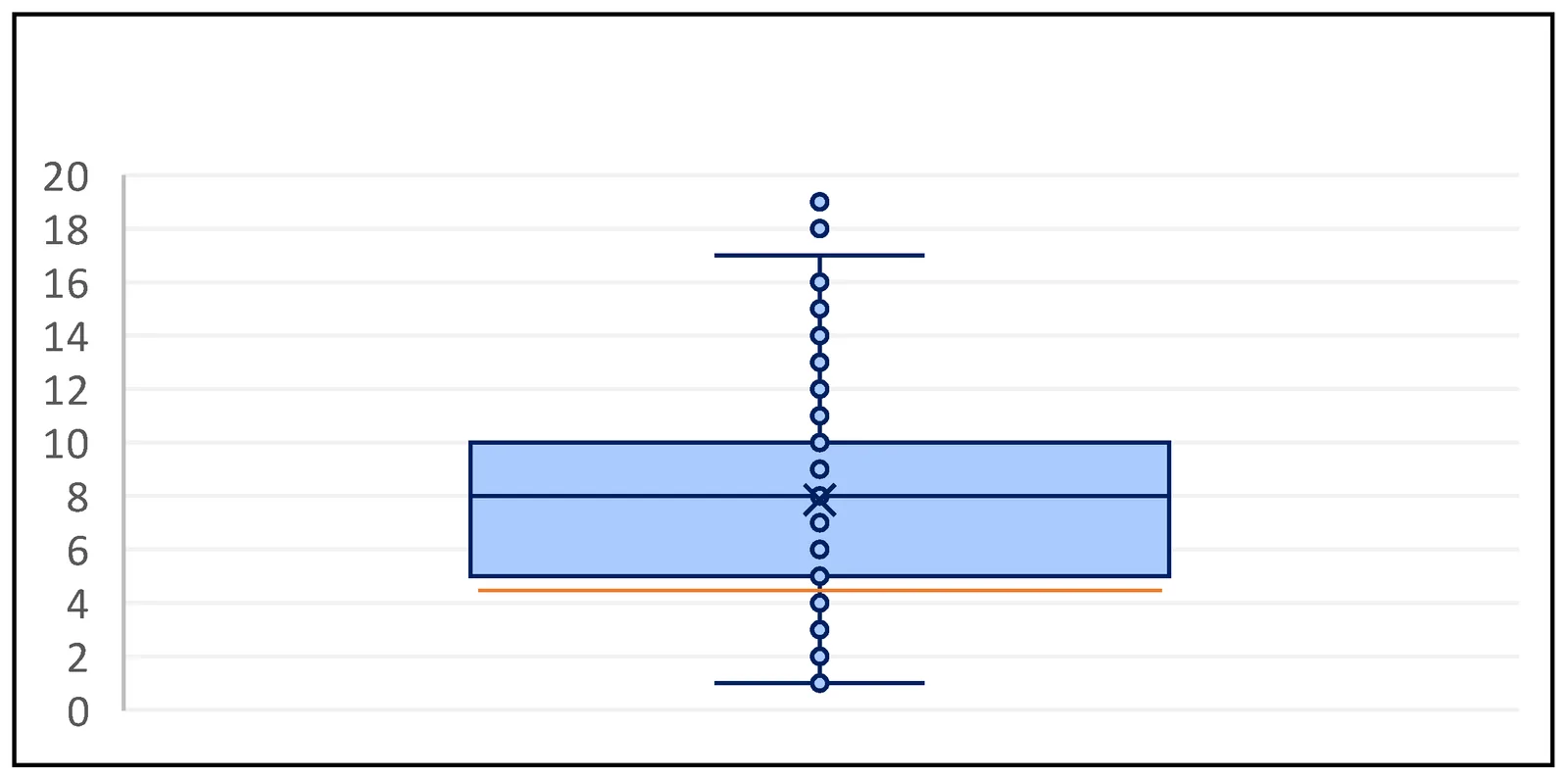
Distribution of global PSQI sleep quality scores (*n* = 265). Boxplot distribution of global PSQI scores. The horizontal line inside the box represents the median (8.0), the “x” indicates the sample mean (7.79 ± 3.53), and the box spans the interquartile range (IQR: 5–10). The red dashed line denotes the standard clinical cutoff threshold (>5.0), where scores above this limit indicate clinically significant poor sleep quality.

At the sample level (*n* = 265), the global PSQI score exhibited a mean of M = 7.79, SD = 3.53. Based on the standard clinical cutoff (PSQI > 5.0), 72.8% of respondents (*n* = 193) were classified as “poor sleepers”, indicating substantial nocturnal disturbance across the student cohort (Figure 2).

The empirical distribution of results spanned nearly the entire measurement spectrum, ranging from a minimum of 0.00 points (reflecting optimal sleep architecture) to a maximum of 19.00 points (indicating severe sleep impairment).

### 3.4. Dietary Habits and Eating Behaviors

A series of Mann–Whitney U tests highlighted significant psychometric variations tied to academic coping behaviors and lifestyle choices.

Energy drink consumption during demanding academic periods was significantly associated with elevated sleep disturbances (Mdn = 9.00 for consumers vs. Mdn = 7.00 for non-consumers, U = 4602.00, z = −3.37, *p* = 0.001, FDR-adjusted q = 0.008 r = 0.21). This coping mechanism also linked to higher levels of problematic dietary patterns, showing positive associations with emotional eating (Mdn = 3.00 vs. Mdn = 2.50, U = 4950.50, z = −2.70, *p* = 0.007, FDR-adjusted q = 0.018, r = 0.17) and uncontrolled eating (Mdn = 2.33 vs. Mdn = 2.22, U = 5331.00, z = −1.97, *p* = 0.048, FDR-adjusted q = 0.076, r = 0.12). No significant group variations emerged for cognitive restraint scores (U = 5511.50, z = −1.63, *p* = 0.103, FDR-adjusted q = 0.147, r = 0.10).

Nicotine consumption patterns similarly tracked with variations in sleep profiles. Specifically, conventional cigarette use was associated with higher global sleep disturbances (Mdn = 9.00) compared to non-smoking status (Mdn = 7.00, U = 4268.50, z = −2.12, *p* = 0.034, FDR-adjusted q = 0.061, r = 0.13). Electronic cigarette smoking was likewise linked to elevated sleep parameters, with users reporting higher sleep disturbance scores than non-users (Mdn = 9.00 vs. Mdn = 7.00, U = 4628.50, z = −2.80, *p* = 0.005, FDR-adjusted q = 0.017, r = 0.17). Notably, neither conventional nor electronic smoking behaviors demonstrated statistically significant relationships with any of the dietary behavior subscales (*p* > 0.05, FDR-adjusted q > 0.05).

Evaluating psychometric parameters based on smoking status demonstrated that active smokers reported significantly higher global sleep disturbances (Mdn = 8.50, IQR = 5.00) compared to non-smokers (Mdn = 7.00, U = 5106.50, z = −2.756, *p* = 0.006, FDR-adjusted q = 0.018, r = 0.17). Conversely, smoking status did not yield statistically significant differences across any of the TFEQ dietary behavior subscales, including cognitive restraint (Mdn = 2.33, IQR = 1.67 vs. Mdn = 2.17, U = 6119.00, z = −0.780, *p* = 0.435, FDR-adjusted q = 0.509, r = 0.05), uncontrolled eating (Mdn = 2.11 vs. Mdn = 2.22, U = 6336.50, z = −0.357, *p* = 0.721, FDR-adjusted q = 0.768, r = 0.02), or emotional eating (Mdn = 2.33, vs. Mdn = 2.17, U = 6203.50, z = −0.616, *p* = 0.538, FDR-adjusted q = 0.607, r = 0.04).

Dietary supplement utilization (e.g., vitamins, lecithin, ginkgo biloba, ginseng) during exams was associated with higher sleep disturbances (Mdn = 9.00 vs. Mdn = 7.00, U = 7024.00, z = −2.40, *p* = 0.016, FDR-adjusted q = 0.036, r = 0.15). Furthermore, supplement use was linked with significantly elevated scores across all dietary subscales, showing higher levels of cognitive restraint (Mdn = 2.83 vs. Mdn = 2.00, U = 5590.00, z = −4.76, *p* < 0.001, FDR-adjusted q = 0.006, r = 0.29), emotional eating (Mdn = 2.67 vs. Mdn = 1.83, U = 6228.00, z = −3.71, *p* < 0.001, FDR-adjusted q = 0.006, r = 0.23), and uncontrolled eating (Mdn = 2.33 vs. Mdn = 2.00, U = 6420.00, z = −3.39, *p* = 0.001, FDR-adjusted q = 0.007, r = 0.21).

The use of smart drugs to maintain attention during exam periods was associated with higher global sleep disturbances (Mdn = 9.00 vs. Mdn = 7.00, U = 2815.50, z = −2.75, *p* = 0.006, FDR-adjusted q = 0.018, r = 0.17). In terms of eating behaviors, cognitive-enhancer use was tied to marginally higher levels of cognitive restraint (Mdn = 2.83 vs. Mdn = 2.17, U = 3039.50, z = −2.21, *p* = 0.027, FDR-adjusted q = 0.050, r = 0.14) and emotional eating (Mdn = 2.67 vs. Mdn = 2.17, U = 2943.00, z = −2.44, *p* = 0.015, FDR-adjusted q = 0.035, r = 0.15), while uncontrolled-eating scores showed no significant variation (U = 3338.50, z = −1.49, *p* = 0.137, FDR-adjusted q = 0.181, r = 0.09).

An increase in coffee consumption during highly demanding academic blocks was likewise associated with elevated sleep disturbances (Mdn = 8.00 vs. Mdn = 7.00, U = 6872.00, z = −2.32, *p* = 0.020, FDR-adjusted q = 0.040, r = 0.14). Increased caffeine intake also tracked with significantly higher scores across all subscales of the eating behavior instrument. It demonstrated positive associations with emotional eating (Mdn = 2.50 vs. Mdn = 1.83, U = 5947.00, z = −3.86, *p* < 0.001, FDR-adjusted q = 0.006, r = 0.24), cognitive restraint (Mdn = 2.50 vs. Mdn = 2.00, U = 6527.50, z = −2.89, *p* = 0.004, FDR-adjusted q = 0.016, r = 0.18), and uncontrolled eating (Mdn = 2.33 vs. Mdn = 2.11, U = 6516.50, z = −2.91, *p* = 0.004, FDR-adjusted q = 0.016, r = 0.18). Overall, these bivariate comparisons indicate significant concurrent associations between increased coffee consumption and other substance-use behaviors.

Regarding dietary regularity, the absence of a regular three-meal schedule was associated with significantly higher scores for emotional eating (Mdn = 2.17 vs. Mdn = 2.00, U = 4379.50, z = −2.77, *p* = 0.006, FDR-adjusted q = 0.018, r = 0.17) and an marginally for uncontrolled eating (Mdn = 2.22 vs. Mdn = 2.00, U = 4754.50, z = −2.02, *p* = 0.043, FDR-adjusted q = 0.072, r = 0.12). No significant group variations were found for global sleep quality (U = 4885.50, z = −1.77, *p* = 0.078, FDR-adjusted q = 0.114, r = 0.11) or cognitive restraint (U = 5584.50, z = −0.38, *p* = 0.706, FDR-adjusted q = 0.764, r = 0.02). Snacking between main meals yielded no statistically significant variations across any evaluated psychometric outcome (*p* > 0.05, FDR-adjusted q > 0.05).

Conversely, daily fruit consumption (at least one fruit per day) emerged as a distinctive factor in relation to both sleep parameters and cognitive dietary regulation. Students reporting daily fruit intake experienced significantly lower global sleep disturbances (Mdn = 7.00 vs. Mdn = 9.00, U = 6294.00, z = −3.98, *p* < 0.001, FDR-adjusted q = 0.006, r = 0.24) alongside higher cognitive restraint scores (Mdn = 2.50 vs. Mdn = 2.17, U = 6822.50, z = −3.13, *p* = 0.002, FDR-adjusted q = 0.009, r = 0.19). Daily fruit intake did not introduce statistically significant shifts in uncontrolled eating (U = 7864.50, z = −1.45, *p* = 0.147, FDR-adjusted q = 0.189, r = 0.09) or emotional eating profiles (U = 8115.50, z = −1.05, *p* = 0.294, FDR-adjusted q = 0.356, r = 0.06).

Evaluating physical activity habits demonstrated that engagement in regular daily physical activity (≥30 min/day) was associated with significantly higher cognitive restraint scores (Mdn = 2.33) compared to sedentary peers (Mdn = 2.17; U = 6536.00, z = −2.399, *p* = 0.016, FDR-adjusted q = 0.036, r = 0.15). Daily physical activity status did not introduce statistically significant shifts regarding global sleep disturbances (Mdn = 8.00 vs. Mdn = 7.00; U = 7614.50, z = −0.581, *p* = 0.562, FDR-adjusted q = 0.619, r = 0.04), uncontrolled eating (U = 7017.00, z = −1.587, *p* = 0.112, FDR-adjusted q = 0.154, r = 0.10), or emotional eating (U = 7430.50, z = −0.890, *p* = 0.373, FDR-adjusted q = 0.444, r = 0.05).

When categorizing participants by exercise intensity (light vs. moderate vs. intense), Kruskal–Wallis H tests established a statistically significant stepwise increase across cognitive restraint scores (H(2) = 14.999, *p* = 0.001, FDR-adjusted q = 0.007, ƞ^2^_H_ = 0.050). Post hoc evaluations confirmed that students performing intense physical activity exhibited the highest intentional dietary monitoring (Mdn = 3.17), followed by those engaging in moderate exercise (Mdn = 2.50) and light activity (Mdn = 2.00).

No significant variation was observed across exercise-intensity levels for global sleep disturbances (Mdn = 8.00 for light; Mdn = 7.00 for moderate; Mdn = 6.00 for intense; H(2) = 5.596, *p* = 0.061, FDR-adjusted q = 0.091, ƞ^2^_H_ = 0.014). No significant variations across activity levels emerged for uncontrolled eating (H(2) = 4.522, *p* = 0.104, FDR-adjusted q = 0.147, ƞ^2^_H_ = 0.010) or emotional eating (H(2) = 3.207, *p* = 0.201, FDR-adjusted q = 0.248, ƞ^2^_H_ = 0.005).

Psychometric eating profiles and sleep quality parameters were systematically evaluated across predominant meal-skipping categories (*n* = 265). Descriptive analysis revealed that breakfast skipping was the most common meal-omission habit (65.7%, *n* = 174), followed by lunch skipping (27.9%, *n* = 74), whereas dinner skipping was practiced by a small minority (6.4%, *n* = 17).

Kruskal–Wallis H tests established statistically significant group variations for uncontrolled eating (H(2) = 7.892, *p* = 0.019, FDR-adjusted q = 0.040, ƞ^2^_H_ = 0.022) and an exploratory variation for cognitive restraint (H(2) = 6.492, *p* = 0.039, FDR-adjusted q = 0.068, ƞ^2^_H_ = 0.017). In contrast, neither emotional eating (H(2) = 4.002, *p* = 0.135, FDR-adjusted q = 0.181, ƞ^2^_H_ = 0.008) nor global sleep disturbances (H(2) = 3.595, *p* = 0.166, FDR-adjusted q = 0.209, ƞ^2^_H_ = 0.006) differed significantly across the three meal-skipping categories.

Post hoc pairwise comparisons using Mann–Whitney U tests confirmed that students who predominantly omitted dinner presented a distinct psychometric profile, characterized by substantially higher cognitive dietary monitoring and reduced dietary disinhibition compared to both breakfast and lunch skippers:

- Cognitive Restraint: Dinner skippers exhibited higher restraint scores (Mdn = 3.00, IQR = 1.67, M ± SD = 2.90 ± 0.92) than breakfast skippers (Mdn = 2.17, IQR = 1.50, M ± SD = 2.31 ± 0.97; U = 943.50, z = −2.467, *p* = 0.014, FDR-adjusted q = 0.036, r = 0.18) as well as compared to lunch skippers (Mdn = 2.42, IQR = 1.37, M ± SD = 2.40 ± 0.90; U = 431.50, z = −2.015, *p* = 0.044, FDR-adjusted q = 0.072, r = 0.21).
- Uncontrolled Eating: Dinner skippers demonstrated lower loss of control over eating (Mdn = 1.67, IQR = 0.72, M ± SD = 1.87 ± 0.45) compared to breakfast skippers (Mdn = 2.22, IQR = 0.92, M ± SD = 2.31 ± 0.73; U = 915.50, z = −2.596, *p* = 0.009, FDR-adjusted q = 0.023, r = 0.19) and lunch skippers (Mdn = 2.22, IQR = 0.81, M ± SD = 2.32 ± 0.63; U = 352.50, z = −2.822, *p* = 0.005, FDR-adjusted q = 0.017, r = 0.30).
- Emotional Eating: Dinner skippers also reported significantly lower affect-driven eating scores (Mdn = 1.67, IQR = 1.08, M ± SD = 1.84 ± 0.68) relative to lunch skippers (Mdn = 2.17, IQR = 1.21, M ± SD = 2.43 ± 0.97; U = 405.00, z = −2.287, *p* = 0.022, FDR-adjusted q = 0.043, r = 0.24).

Notably, no statistically significant differences emerged when comparing breakfast skippers directly to lunch skippers across any evaluated psychometric dimension (*p* > 0.05). Global PSQI sleep scores remained consistently elevated across all subgroups, ranging from Mdn = 7.00 (M ± SD = 7.22 ± 3.22) for lunch skippers to Mdn = 8.00 (M ± SD = 8.08 ± 3.69) for breakfast skippers, reaffirming a widespread pattern of poor sleep quality across the overall student population regardless of meal habits.

Cross-tabulation analyses using Pearson’s chi-square (chi^2^) tests demonstrated significant gender disparities regarding academic coping behaviors and health perceptions. Male students were significantly more likely to consume energy drinks during examination sessions compared to female peers (40.3% vs. 19.6%; chi^2^ (1) = 11.422, *p* < 0.001, FDR-adjusted q = 0.006). Conversely, male respondents also exhibited a higher rate of positive health self-appraisal, with 54.4% rating their overall lifestyle as healthy compared to only 38.1% of female respondents (chi^2^ (1) = 5.532, *p* = 0.019, FDR-adjusted q = 0.040).

Evaluation of concurrent academic coping strategies revealed a distinct pattern of multi-substance reliance centered around increased caffeine intake during examination periods. Students who reported increasing their coffee consumption during exams were significantly more likely to concurrently utilize energy drinks (34.6% vs. 11.1%; chi^2^ (1) = 18.742, *p* < 0.001, FDR-adjusted q = 0.006), general dietary supplements (48.4% vs. 31.5%; chi^2^ (1) = 7.441, *p* = 0.006, FDR-adjusted q = 0.018), and smart drugs (19.0% vs. 5.6%; chi^2^ (1) = 9.788, *p* = 0.002, FDR-adjusted q = 0.009). OTC supplement use did not differ significantly between coffee-increasing students and non-increasers (58.2% vs. 46.3%; chi^2^ (1) = 3.585, *p* = 0.058, FDR-adjusted q = 0.089).

A marginally statistically significant association was identified regarding daily fruit consumption (≥1 fruit/day), with urban students exhibiting a higher rate of daily fruit intake compared to their rural peers (53.0% vs. 40.6%; chi^2^ (1) = 3.883, *p* = 0.049, FDR-adjusted q = 0.080).

Conversely, no significant residential disparities were observed regarding weekly fast-food consumption frequency (chi^2^ (3) = 1.146, *p* = 0.766, FDR-adjusted q = 0.803), weekly carbonated soft drink intake (chi^2^ (3) = 2.069, *p* = 0.558, FDR-adjusted q = 0.619), or energy drink reliance during exam sessions (30.5% urban vs. 26.7% rural; chi^2^ (1) = 0.428, *p* = 0.513, FDR-adjusted q = 0.589), indicating homogeneous consumption patterns for processed foods and caffeine-based beverages across residential origins).

### 3.5. Regression Analysis

The combined multivariable association of sleep quality, dietary behaviors, and age with the likelihood of using energy drinks during examination periods was evaluated via a binary logistic regression model. A total of *n* = 261 valid cases were included in the multivariate model (four cases excluded due to missing predictors). The multivariable model was statistically significant, with an overall classification accuracy of 75.5% (sensitivity = 6.2%, specificity = 98.5%) and acceptable discrimination (AUC = 0.662, 95% CI [0.586, 0.739], *p* < 0.001). The Hosmer–Lemeshow test indicated adequate calibration for the baseline behavioral predictors (chi^2^ (8) = 9.750, *p* = 0.283). The full parameter estimates and statistical indicators for this regression model are detailed in Table 4.

Within this multivariate arrangement, global sleep quality emerged as a statistically significant independent correlate of energy drink use. Every one-point increase in the PSQI Global Score (indicating worsening sleep quality) was associated with an 11.8% increase in the odds of consuming energy drinks (B = 0.111, SE = 0.046, Wald = 5.980, *p* = 0.014, OR = 1.118, 95% CI [1.022, 1.222]).

Additionally, age demonstrated a statistically significant inverse association with energy drink consumption (B = −0.171, SE = 0.070, Wald = 5.938, *p* = 0.015, OR = 0.843, 95% CI [0.735, 0.967]). This indicates that each additional year of age was linked to 15.7% lower odds of using energy drinks during examination periods, highlighting higher consumption patterns among younger students.

When controlling for sleep quality and age, none of the specific eating behavior subscales—cognitive restraint (*p* = 0.177), uncontrolled eating (*p* = 0.678), and emotional eating (*p* = 0.266)—showed independent associations with energy drink usage. These findings demonstrate that poor sleep quality and younger age are the primary factors associated with energy drink reliance during academic examination phases.

A binary logistic regression model was estimated to determine how dietary behaviors, physical activity, and smoking habits are jointly associated with students’ self-appraisal of maintaining a healthy lifestyle (*n* = 265). The multivariate model demonstrated robust statistical significance, chi^2^ (7) = 67.171, *p* < 0.001, accounting for 30.1% of the total variance in self-perceived health status (Nagelkerke R^2^ = 0.301; Cox and Snell R^2^ = 0.224). The predictive model achieved an overall classification accuracy of 72.1%, with satisfactory goodness of fit as confirmed by the Hosmer and Lemeshow test (chi^2^ (8) = 2.762, *p* = 0.948). The parameter estimates and fit metrics are detailed in Table 5.

Empirical findings highlight daily fruit intake as the strongest positive correlate of health self-perception. Respondents consuming at least one fruit per day exhibited nearly a fourfold increase in the likelihood of rating their lifestyle as healthy compared to non-consumers (B = 1.377, SE = 0.294, Wald = 21.962, *p* < 0.001, OR = 3.962, 95% CI [2.228, 7.047]). Adherence to a structured three-meal daily schedule similarly tracked with positive health self-appraisals, nearly tripling the odds of a favorable self-assessment (B = 1.073, SE = 0.353, Wald = 9.240, *p* = 0.002, OR = 2.924, 95% CI [1.464, 5.841]).

Unfavorable dietary patterns, sedentary behavior, and conventional tobacco smoking were significantly associated with lower odds of favorable healthy-lifestyle self-identification. Higher weekly fast-food consumption frequency was associated with 40.0% lower odds of reporting a healthy lifestyle (B = −0.511, SE = 0.214, Wald = 5.705, *p* = 0.017, OR = 0.600, 95% CI [0.394, 0.912]). Active conventional cigarette smoking was associated with 59.9% lower odds of positive health self-appraisal (B = −0.914, SE = 0.403, Wald = 5.127, *p* = 0.024, OR = 0.401, 95% CI [0.182, 0.884]).

Furthermore, physical inactivity (failing to complete at least 30 min of daily exercise) was associated with a 49.6% reduction in the odds of a favorable lifestyle rating (B = −0.686, SE = 0.319, Wald = 4.635, *p* = 0.031, OR = 0.504, 95% CI [0.270, 0.940]). Neither electronic cigarette use (*p* = 0.230) nor carbonated soft drink intake (*p* = 0.741) demonstrated independent statistical significance within the adjusted model.

### 3.6. Cluster Analysis and Behavioral Typologies

To evaluate whether academic coping behaviors manifest as distinct patterns during examination periods, a TwoStep cluster analysis was conducted on binary consumption indicators (coffee escalation, energy drinks, dietary supplements, OTC vitamins, and smart drugs). The model revealed three distinct, well-balanced student typologies (N = 261 valid cases, 4 missing) with satisfactory internal cohesion (silhouette measure of cohesion and separation = 0.38):

1. Cluster 1: Non-users (*n* = 85, 32.6%), representing students with minimal reliance on chemical substances during examinations (0.0% energy drinks, 0.0% supplements, 0.0% smart drugs, and 44.7% increased coffee intake).
2. Cluster 2: Poly-substance users (*n* = 90, 34.5%), characterized by intensive concurrent substance reliance, encompassing all reported energy drink users (72.2%) and all smart-drug consumers (38.9%), alongside high rates of coffee escalation (77.8%) and dietary supplement intake (58.9%).
3. Cluster 3: OTC Supplement and caffeine users (*n* = 86, 33.0%), defined by universal over-the-counter vitamin and supplement utilization (100.0%) and moderate coffee increase (52.3%), in the complete absence of energy drinks (0.0%) or smart drugs (0.0%).

Kruskal–Wallis H tests established highly statistically significant differences across all evaluated psychometric eating dimensions and global sleep scores across the three behavioral typologies (all *p* < 0.001, FDR-adjusted q = 0.006; Table 6).

Non-users (Cluster 1) consistently demonstrated the lowest mean rank scores across all evaluated psychometric eating dimensions and global sleep disturbances. In contrast, poly-substance users (Cluster 2) exhibited the highest global sleep disturbances (PSQI mean rank = 157.20 vs. 106.69 for Cluster 1, H(2) = 19.981, *p* < 0.001) and the highest levels of emotional eating (mean rank = 151.46 vs. 99.64, H(2) = 22.779, *p* < 0.001) and uncontrolled eating (mean rank = 144.74 vs. 103.79, H(2) = 16.446, *p* < 0.001).

### 3.7. Ethical Perspectives and the Consumption of Smart Drugs

To examine the correlations between the psychometric scales, ethical perceptions and demographic variables, Spearman’s rank-order correlation (r_s_) and chi-square (chi^2^) tests of independence were utilized.

Regarding ethical perceptions and academic pressure, the belief that academic pressure justifies smart-drug use displayed a significant negative correlation with global sleep disturbances (r_s_ = −0.185, *p* = 0.003, FDR-adjusted q = 0.012) and an exploratory correlation with the perception that cognitive enhancement is unethical (r_s_ = 0.132, *p* = 0.033, FDR-adjusted q = 0.060).

Conversely, ethical attitudes toward smart drugs showed no statistically significant relationships with any of the three TFEQ eating behavior dimensions (*p* > 0.05, FDR-adjusted q > 0.05), indicating that moral frameworks regarding smart drugs operate independently of psychological eating patterns.

A binary logistic regression model was conducted to evaluate the combined association of academic pressure justification, ethical perceptions, and professional responsibilities with the likelihood of using smart drugs during examination periods (*n* = 261, including 35 positive events, EPV = 11.67; Table 7). The multivariable model was statistically significant, chi^2^ (3) = 28.303, *p* < 0.001, explaining 18.8% of the variance in smart-drug utilization (Nagelkerke R2 = 0.188; Cox and Snell R^2^ = 0.103). The model yielded an overall correct classification rate of 86.6%, which was driven by high specificity (100.0%) and low sensitivity (0.0%) due to outcome class imbalance. Importantly, the model demonstrated good discriminative ability, as evidenced by an Area Under the ROC Curve (AUC) of 0.777 (95% CI [0.705, 0.849], *p* < 0.001), alongside a significant Hosmer–Lemeshow goodness-of-fit statistic (chi^2^ (8) = 21.447, *p* = 0.006). The parameter estimates and statistical indicators for this regression model are detailed in Table 7.

The perception regarding academic pressure justification was independently associated with smart-drug consumption during examinations (Table 7). Because the attitude scale was coded from 1 (“Strongly Agree”) to 5 (“Strongly Disagree”), each one-unit increase toward disagreement was associated with a 50.6% reduction in the odds of using smart drugs (B = −0.705, SE = 0.143, Wald = 24.346, *p* < 0.001, OR = 0.494, 95% CI [0.373, 0.654]). Inversely, stronger personal agreement that academic demands justify cognitive enhancement was significantly associated with higher odds of substance use. Neither general ethical views (*p* = 0.652) nor perceived professional duties (*p* = 0.764) retained independent statistical significance.

## 4. Discussion

A poor global sleep score was identified among respondents. Higher global sleep disturbance scores were associated with the use of dietary supplements, such as vitamins, lecithin, ginkgo biloba, and ginseng, as well as increased coffee consumption, energy drink intake, and the use of smart drugs. Academic pressure justification primarily correlates with smart-drug use, with a strong, statistically significant negative relationship indicating that personal coping mechanisms override general ethical or professional considerations.

Active smokers also reported significantly higher global sleep disturbance scores, with conventional cigarette use showing a significant association with poorer sleep quality. Our data are similar to those identified among healthcare students in other countries: in Türkiye, Avcu et al. [28] found that smoking was associated with reduced sleep quality, while in France daily smokers were more frequently short sleepers than occasional smokers and non-smokers [29].

An interesting result of the present study concerns tobacco use: while conventional smoking was negatively associated with students’ self-perceived health, electronic cigarette use showed no independent association in the multivariable model. Although psychological constructs such as risk perception or cognitive disconnect were not directly measured in our survey, one speculative interpretation supported by the prior literature is that students who vape do not perceive this behavior as incompatible with a healthy lifestyle, possibly because e-cigarettes are often marketed as a cleaner alternative to conventional smoking [14]. This potential behavioral explanation remains hypothetical and warrants direct psychological evaluation in future studies.

The analysis of answers revealed that 58.6% of students reported a perceived increase in coffee consumption during exam season, 24.9% reported using energy drinks and 41.4% dietary supplements, 53.3% declared that they use OTC supplements (vitamins, lecithin, ginkgo biloba, ginseng) and 13.4% reported they use smart drugs during evaluation periods. Male students were significantly more likely to consume energy drinks during examination periods compared to female peers. Multivariate analysis indicates that poor sleep quality, marked by high PSQI scores, is independently associated with higher odds of energy drink use (OR = 1.118), while younger age is inversely associated with consumption (OR = 0.843) compared to older peers. Furthermore, logistic modeling shows that student attitudes—specifically academic pressure, ethical perceptions, and professional responsibilities—are robustly associated with smart-drug use, explaining 18.8% of the variance.

Interestingly, students who reported increasing their coffee consumption during exams were significantly more likely to concurrently utilize energy drinks, general dietary supplements and smart drugs. OTC supplement use was also descriptively evaluated alongside other stimulant behaviors. Students who reported increasing their coffee consumption during examination periods appear more likely to engage in self-reported concurrent stimulant and supplement use as an exam-specific coping mechanism aimed at enhancing academic performance, rather than relying on coffee alone. Nearly one in five students who reported increased coffee intake also acknowledged using smart drugs. These findings suggest that higher coffee consumption may serve as a behavioral indicator of students actively seeking external chemical strategies to cope with academic pressure. This pattern may involve combining everyday caffeine intake with energy drinks, dietary supplements, and smart drugs. Such combined use may increase the risk of adverse effects, including excessive caffeine intake, anxiety, and sleep disruption, situating the students in a vicious circle.

Similar findings were identified in a study in the UAE; Al Ghali et al. [30] assessed the prevalence and perceived benefits of caffeinated beverage consumption among Emirati university students, finding a large majority consumed caffeine (98.5%), of whom almost one third (31.0%) reported being addicted to caffeine. In Korea, the most consumed caffeine beverages for increasing academic performance during exam season were coffee (79.2%), soda (33.2%), tea (27.4%), chocolate (25.2%), and energy drinks (20.5%) in line with the findings of Choi et al. [31]. In Saudi Arabia, El Seifi et al. [32] identified that 81.3% of medical students reported consuming caffeine and 80.6% reported some negative effects. In Jordan, coffee consumption was associated with energy drinks and alcohol consumption [33]. In Malta, the study of Vella-Fondacaro et al. [34] showed that high levels of coffee and energy drink consumption are found among medical students, with no difference present when comparing to junior doctors. Opposite results were also identified. For example, in India, the prevalence of caffeine intake was 85.7%, where tea was the most consumed while coffee and energy drinks were least consumed by healthcare students [35].

Our study highlights that daily fruit intake is associated with students’ health self-perception. Respondents who consumed at least one fruit per day were nearly four times more likely to rate their lifestyle as healthy than non-consumers. Students reporting daily fruit intake also experienced significantly lower global sleep disturbances.

Our results are similar to other studies. In Poland, Kosendiak et al. [19] showed that the use of sleep medications was highest among healthcare students with insufficient nutritional knowledge. Using a pre- and post-test design, the researchers identified that, throughout the academic year, consumption increased for all except those with the highest nutritional knowledge, who also exhibited the best sleep. Dieting, exercise and sleep are appreciated to be key pillars of a healthy lifestyle for medical students [36].

The present research identified that engagement in regular daily physical activity was associated with significantly higher cognitive restraint scores compared to sedentary peers. Although neurocognitive mechanisms, stress reduction, and impulse control were not directly evaluated in our protocol, the previous literature suggests that regular physical activity may positively support self-regulation and inhibitory functioning [37,38,39]. These literature-based pathways represent plausible theoretical explanations for the observed association rather than mechanisms confirmed by our cross-sectional data. Physical activity levels varied considerably across countries. In Saudi Arabia, only 4.3% of medical students reported engaging in more than 30 min of physical activity per day [40]. In Poland, many students reported moderate or vigorous levels of physical activity [41]. By contrast, in Norway, 91% of participants reported engaging in at least four hours of physical activity per week [42].

Regarding attitudes toward smart drugs, our findings demonstrate that personal justification under academic pressure was strongly associated with smart-drug consumption during examination periods. Because the item was coded from 1 (Strongly Agree) to 5 (Strongly Disagree), the observed negative coefficient (OR = 0.494) confirms that students expressing disagreement had significantly lower odds of substance use, whereas those who endorsed academic pressure as an acceptable rationale were substantially more likely to use smart drugs. This aligns with previous evidence indicating that personal coping rationalizations under acute academic stress frequently outweigh abstract ethical considerations or professional norms among healthcare students [43,44].

A TwoStep cluster analysis identified three distinct, well-balanced student typologies during exams: non-users (32.6%), poly-substance users (34.5%), and OTC supplement/caffeine users (33.0%), demonstrating that multi-substance coping is closely linked to higher nocturnal sleep disturbance and maladaptive eating patterns. This balanced distribution showed that the typologies are distinct and representative. This equal distribution proved that multi-substance consumption is not an isolated behavior but a coping paradigm that affected more than one third of medical students. The anaysis provided profound insights into how university students navigate acute academic stress. The poly-substance user profile demonstrated the strongest statistical association with severe nocturnal sleep disturbances (measured by the PSQI) and maladaptative eating patterns (evaluated with TFEQ-21) indicating that this coping mechanism escalated from benign or single-substance use (such as caffeine) to multi-substance use, creating a physiological loop: this practice disrupts nocturnal sleep architecture and causes sleep deficit, in turn inducing fatigue that drives emotional or uncontrolled eating patterns.

The cluster analysis conducted in this study demonstrated that examining caffeine use, smart-drug consumption or sleep patterns in isolation would have overlooked the extent to which these behaviors are structurally interconnected within three distinct student profiles.

These results show that medical students have a heightened need to stay alert during examination periods, enabling them to cope with the large volume of theoretical information doubled by the practical internships they have to prepare for. It is very possible that this result actually reflects a specific culture of medical students (large volume of information, academic stress and fear of failure in an environment where you work with human subjects and where you are not allowed to make mistakes), encouraged by knowledge of the effects of substance use on the brain.

### 4.1. Strengths and Limitations of the Study

The study successfully integrates three distinct behavioral domains: substance and stimulant use, including energy drinks, smart drugs, coffee, and smoking; dietary psychology, assessed through the validated Three-Factor Eating Questionnaire (TFEQ); and lifestyle habits, including sleep patterns, physical activity, and meal skipping. By combining these dimensions, the study provides a comprehensive academic coping framework for understanding students’ health-related behaviors during periods of high academic demand. Rather than relying exclusively on simple correlational analyses, the study employs advanced multivariate binary logistic regression models. These models allow for the control of potential confounding variables and enable the estimation of precise risk increments through odds ratios.

This study has several methodological limitations that warrant careful consideration:

First, it relies on a cross-sectional design, which limits the identification of causality, temporal sequences, or directionality between academic examination stress, sleep disruption, and compensatory eating or substance-use mechanisms. Additionally, because baseline non-exam measurements were not longitudinally tracked, items assessing “increased consumption” represent subjective, self-reported perceptions of behavioral adaptation rather than prospective, objectively verified changes relative to a baseline. Second, data collection was based entirely on self-administered scales, which introduces potential subjectivity, recall bias, and social desirability effects, particularly regarding sensitive behaviors such as smart-drug consumption. Third, recruitment was conducted via a non-probability snowball sampling method through digital peer channels across two Romanian academic centers. Because the invitation link was openly disseminated, the exact denominator of exposed students could not be objectively tracked, preventing the calculation of a formal response rate and introducing potential selection bias (e.g., self-selection by students experiencing greater academic strain or possessing higher health awareness). Fourth, although the sample included participants from two universities (Iași and Oradea), the findings cannot be generalized to the broader university population. The cohort exhibited a high proportion of female participants (74.3%) and a predominance of preclinical students (80.4% in Years 1–3 vs. 18.1% in Years 4–6), which may reflect gender-related survey compliance and potentiates residual confounding related to varying clinical training workloads. Fifth, the high classification accuracy of the logistic regression models reflects high specificity due to class imbalance, as confirmed by low sensitivity and moderate AUC values (AUC = 0.662 for energy drinks and AUC = 0.777 for smart drugs), highlighting the need for cautious interpretation of individual predictive classifications. Additionally, although the smart-drug model met the EPV ≥ 10 stability guideline (35 events, 3 predictors), the relatively modest absolute number of positive cases warrants cautious interpretation until validated in larger cohorts. Sixth, the study performed multiple bivariate comparisons across dietary habits, substance coping, and psychometric dimensions. Testing numerous unadjusted significance hypotheses increases the family-wise risk of Type I error (false-positive findings). To address this, multivariable logistic regression was utilized as our primary confirmatory framework, and the Benjamini–Hochberg False Discovery Rate (FDR) procedure was applied across all 66 bivariate tests. While our core findings remained fully robust (q < 0.05), marginally significant bivariate findings (q = 0.050–0.080) are explicitly characterized as exploratory trends that require verification in future confirmatory cohorts. Additionally, while statistically significant after FDR adjustment, the observed bivariate effect sizes were predominantly small to moderate (r = 0.12–0.30; ƞ^2^_H_ = 0.01–0.08), reflecting the multifactorial nature of academic stress and lifestyle adaptations, which reinforces the importance of interpreting these relationships within multivariable frameworks rather than in isolation. Seventh, the global PSQI demonstrated a modest internal consistency (α = 0.683), falling slightly below the conventional 0.70 benchmark; while the scale remains widely used and validated in university populations, composite sleep quality scores should be interpreted with appropriate caution. Eighth, behavioral typologies were identified via cluster analysis on binary indicators; although cluster cohesion was verified, these profiles lack external validation in an independent student cohort. Additionally, items measuring situational academic coping and ethical perceptions were study-specific rather than standardized psychometric instruments. Finally, residual confounding cannot be ruled out regarding unmeasured variables such as objective academic workload, socioeconomic background, and baseline psychological distress or generalized anxiety.

### 4.2. Practical Implications

The findings provide practical insights for university administrators, student health services departments and academic policymakers. Not limited to descriptive statistical data, these results shape institutional interventions aimed at reducing the maladaptive behaviors of healthcare students during the examination periods.

First, the multivariate analysis indicated that younger age is independently associated with energy drink use. Considering the numerous negative physical and psychological correlates of this kind of consumption, the result suggests that freshman students may exhibit greater vulnerability to coping behavior than older students.

Second, sleep disruption appears to function as a central component of multi-substance coping patterns, involving energy drinks, smart drugs and coffee. This result highlights the need to identify behaviors that may mask sleep-related disorders rather than focusing exclusively on anxiety or academic stress.

Third, the study identifies a statistically significant stepwise increase in cognitive dietary monitoring across levels of physical intensity alongside observations regarding sleep quality across exercise-intensity levels. This result suggests that university campus administrators should facilitate both relaxation and sports facilities during examination periods.

Fourth, students seem to perceive themselves as having healthy behaviors if they eat fruit and do not perceive the risk of e-cigarette use. This result suggests the need for better information on multifaceted healthy-diet behavior and the negative impact of e-cigarette use on their health.

## 5. Conclusions

The present cross-sectional study highlights that healthcare university students report distinct, interrelated behavioral patterns characterized by co-occurring poor sleep quality, irregular dietary routines, and compensatory use of stimulants or related substances in the context of academic examination periods. Given the observational nature of the study and the absence of a non-examination comparison baseline, these findings reflect cross-sectional associations rather than causal effects of examination stress. While the present observational data do not evaluate or demonstrate the efficacy of specific programs, these identified patterns provide useful context to inform future institutional health promotion strategies and support mechanisms for healthcare students facing demanding academic phases.

## Figures and Tables

**Table 1:** Health-related behavior and lifestyle characteristics of the sample (*n* = 265) ^1^.

Characteristics	M ± SD or *n* (%)
Sex	
Female	197 (74.3%)
Male	68 (25.7%)
Residential Origin	
Urban	164 (61.9%)
Rural	101 (38.1%)
Perceived Healthy Lifestyle—yes	112 (42.3%)
Regular 3 Meals/Day—yes	55 (20.8%)
Daily Fruit Consumption (≥1/day)—yes	128 (48.3%)
Snacking Between Main Meals—yes	208 (78.5%)
Fast-Food Consumption (per week)	
Once	187 (70.6%)
Twice	51 (19.2%)
Three times	16 (6.0%)
More than 3 times	11 (4.2%)
Carbonated Soft Drinks (per week)	
Once	145 (54.7%)
Twice	45 (17.0%)
Three times	28 (10.6%)
More than 3 times	47 (17.7%)
Smoking Status	
Non-smoker	163 (61.5%)
Former smoker	22 (8.3%)
Active smoker	80 (30.2%)
Conventional Cigarette Use—yes	49 (18.5%)
Electronic Cigarette Use—yes	59 (22.3%)
Smart-Drug Use—yes	14 (5.3%)
Daily Physical Activity (30 min) —yes	173 (65.3%)
Predominant Physical Activity Type	
Light	150 (56.6%)
Moderate	96 (36.2%)
Intense	19 (7.2%)
Alcohol Consumption	
Never	88 (33.2%)
Rarely	105 (39.6%)
Sometimes	63 (23.8%)
Quite often	6 (2.3%)
Frequently	3 (1.1%)
Interest in Nutritional Education Programs—yes	175 (66.0%)

^1^ Data are presented as mean ± standard deviation (M ± SD) for continuous variables and as absolute frequencies (*n*) and valid percentages (%) for categorical variables. Percentages for conventional and electronic cigarette use do not sum to the total active smoker percentage because categories are not mutually exclusive (accounting for dual users).

**Table 2:** Data about consumption of coffee, energy drinks and supplements during examination periods (*n* = 261) ^1^.

Characteristics	M ± SD or *n* (%)
Increased coffee consumption during exam season	153 (58.6%)
Increased energy drink consumption (in exam season)	65 (24.9%)
General dietary supplement consumption (in exam season)	108 (41.4%)
OTC supplement use (vitamins, lecithin, ginkgo biloba, ginseng)	139 (53.3%)
Smart-drug use (in exam season)	35 (13.4%)
Academic pressure justifies smart-drug use (1–5 Likert)	3.61 ± 1.39
Strongly disagree	33 (12.6%)
Disagree	21 (8.0%)
Neutral/Uncertain	60 (23.0%)
Agree	47 (18.0%)
Strongly agree	100 (38.3%)
Smart-drug use is unethical (1–5 Likert)	3.74 ± 1.24
Strongly disagree	19 (7.3%)
Disagree	15 (5.7%)
Neutral/Uncertain	82 (31.4%)
Agree	43 (16.5%)
Strongly agree	102 (39.1%)
Medical students should avoid substance abuse/dependence (1–5 Likert)	2.17 ± 1.24
Strongly disagree	112 (42.9%)
Disagree	44 (16.9%)
Neutral/Uncertain	71 (27.2%)
Agree	16 (6.1%)
Strongly agree	18 (6.9%)

^1^ *n* = 261 valid cases after excluding 4 cases with missing data on specific predictor/coping items. Data are presented as mean ± standard deviation (M ± SD) for continuous variables and as absolute frequencies (*n*) and valid percentages (%) for categorical variables.

**Table 3:** Internal consistency, descriptive statistics, and severity distribution for TFEQ-R21 subscales (*n* = 265; *n*/%) ^1^.

Subscale	Cronbach’s α	M ± SD	Low	Moderate	High
Cognitive Restraint	0.910	2.38 ± 0.96	140 (52.8%)	101 (38.1%)	24 (9.1%)
Uncontrolled Eating	0.879	2.28 ± 0.70	169 (63.8%)	82 (30.9%)	14 (5.3%)
Emotional Eating	0.948	2.33 ± 1.07	154 (58.1%)	80 (30.2%)	31 (11.7%)

^1^ Subscales were calculated on a standardized 1.00–5.00 rating scale. Low (1.00–2.33), moderate (2.34–3.66), and high (3.67–5.00) brackets represent study-specific descriptive tertile intervals of the 1–5 scale. M (mean), SD (standard deviation), *n* (number), % (percentages).

**Table 4:** Logistic regression model parameters for evaluating factors associated with energy drink consumption during exam sessions (*n* = 261) ^1^.

Predictor Variables	B	S.E.	Wald	*df*	*p*	OR (Exp B)	95% CI for OR
Global Sleep Quality (PSQI)	0.111	0.046	5.980	1	0.014	1.118	1.022–1.222
Cognitive Restraint Score	0.224	0.166	1.824	1	0.177	1.251	0.904–1.731
Uncontrolled-Eating Score	0.128	0.309	0.172	1	0.678	1.137	0.621–2.082
Emotional Eating Score	0.230	0.207	1.239	1	0.266	1.258	0.839–1.887
Age	−0.171	0.070	5.938	1	0.015	0.843	0.735–0.967

^1^ *n* = 261 valid cases after excluding 4 cases with missing data on specific predictor/coping items, B = unstandardized regression coefficient; S.E. = standard error; Wald = Wald test statistic; *p* = statistical significance level; OR = odds ratio; CI = confidence interval.

**Table 5:** Logistic regression model parameters for identifying factors associated with self-perceived healthy-lifestyle status (*n* = 265) ^1^.

Predictor Variables	B	S.E.	Wald	*df*	*p*	OR (Exp B)	95% CI for OR
Daily Fruit Consumption (≥1/day)	1.377	0.294	21.962	1	<0.001	3.962	2.228–7.047
Regular 3 Meals/Day	1.073	0.353	9.240	1	0.002	2.924	1.464–5.841
Fast-Food Consumption Frequency	−0.511	0.214	5.705	1	0.017	0.600	0.394–0.912
Conventional Cigarette Smoking	−0.914	0.403	5.127	1	0.024	0.401	0.182–0.884
Lack of Daily Physical Activity (<30 min/day)	−0.686	0.319	4.635	1	0.031	0.504	0.270–0.940
Electronic Cigarette Smoking	−0.437	0.364	1.440	1	0.230	0.646	0.316–1.319
Carbonated Soft Drinks Frequency	0.045	0.135	0.109	1	0.741	1.046	0.803–1.362

^1^ B = unstandardized regression coefficient; S.E. = standard error; Wald = Wald test statistic; *p* = statistical significance level; OR = odds ratio; CI = confidence interval.

**Table 6:** Kruskal–Wallis H test comparisons across TwoStep behavioral clusters (*n* = 261) ^1^.

Scale	Cluster 1 (*n* = 85) Mean Rank	Cluster 2 (*n* = 90) Mean Rank	Cluster 3 (*n* = 86) Mean Rank	Kruskal–Wallis H	*df*	*p*-Value	Effect Size (ƞ^2^_H_)
Cognitive Restraint	106.57	144.99	140.50	13.411	2	0.001	0.044
Uncontrolled Eating	103.79	144.74	143.51	16.446	2	<0.001	0.056
Emotional Eating	99.64	151.46	140.59	22.779	2	<0.001	0.081
PSQI Global Score	106.69	157.20	127.60	19.981	2	<0.001	0.070

^1^ *n* = 261 valid cases after excluding 4 cases with missing data on specific predictor/coping items.

**Table 7:** Logistic regression model parameters for evaluating variables associated with smart-drug consumption during exam sessions (*n* = 261) ^1^.

Predictor Variables	B	S.E.	Wald	*df*	*p*	OR (Exp B)	95% CI for OR
Academic pressure justifies smart drugs	−0.705	0.143	24.346	1	<0.001	0.494	0.373–0.654
Cognitive enhancement is unethical	0.067	0.150	0.203	1	0.652	1.070	0.798–1.434
Medical students should avoid substance abuse/dependence	−0.046	0.155	0.090	1	0.764	0.955	0.705–1.293

^1^ *n* = 261 valid cases after excluding 4 cases with missing data on specific predictor/coping items, B = unstandardized regression coefficient; S.E. = standard error; Wald = Wald test statistic; *p* = statistical significance level; OR = odds ratio; CI = confidence interval.

## Data Availability

Data Availability Statements are available upon request from the main author.

## Abbreviations

The following abbreviations are used in this manuscript:

B	Unstandardized regression coefficient
CI	Confidence interval
*dfs*	Degrees of freedom
Exp(B)	Exponentiated coefficient (odds ratio)
M	Mean
Mdn	Median
Min	Minimum
Max	Maximum
N	Number of participants/sample size
OR	Odds ratio
*p*	Statistical significance level
r	Non-parametric effect size for Mann–Whitney U test
ƞ^2^_H_	Eta-squared effect size for Kruskal–Wallis H test
r_s_	Spearman’s rank correlation coefficient
R^2^	Coefficient of determination (Nagelkerke/Cox and Snell pseudo-R-squared)
SD	Standard deviation
SE	Standard error
U	Mann–Whitney U test statistic
Wald	Wald test statistic
z	Standardized Z-score (Mann–Whitney/logistic regression indicator)
chi^2^	Chi-square statistics (model fit indicator)
